# Cross-immunity and age patterns of influenza A(H5N1) infection

**DOI:** 10.1017/S0950268814001976

**Published:** 2014-08-13

**Authors:** A. J. KUCHARSKI, W. J. EDMUNDS

**Affiliations:** Department of Infectious Disease Epidemiology, London School of Hygiene & Tropical Medicine, London, UK

**Keywords:** Avian flu, emerging infections, epidemiology, influenza A, mathematical modelling

## Abstract

The age distribution of influenza A(H5N1) cases reported during 2006–2013 varied substantially between countries. As well as underlying demographic profiles, it is possible that cross-immunity contributed to the age distribution of reported cases: seasonal influenza A(H1N1) and avian influenza A(H5N1) share the same neuraminidase subtype, N1. Using a mechanistic model, we measured the extent to which population age distribution and heterosubtypic cross-immunity could explain the observed age patterns in Cambodia, China, Egypt, Indonesia and Vietnam. Our results support experimental evidence that prior infection with H1N1 confers partial cross-immunity to H5N1, and suggest that more than 50% of spillover events did not lead to reported cases of infection as a result. We also identified age groups that have additional risk factors for influenza A(H5N1) not captured by demography or infection history.

## INTRODUCTION

The potential for highly pathogenic infections to transmit from animals to humans is a major public health concern [1, 2]. Between 1 January 2006 and 1 December 2013, a total of 500 confirmed influenza A(H5N1) cases were reported to the World Health Organization. Of these, 467 cases came from five countries: Cambodia, China, Egypt, Indonesia and Vietnam.

**Fig. 1.**
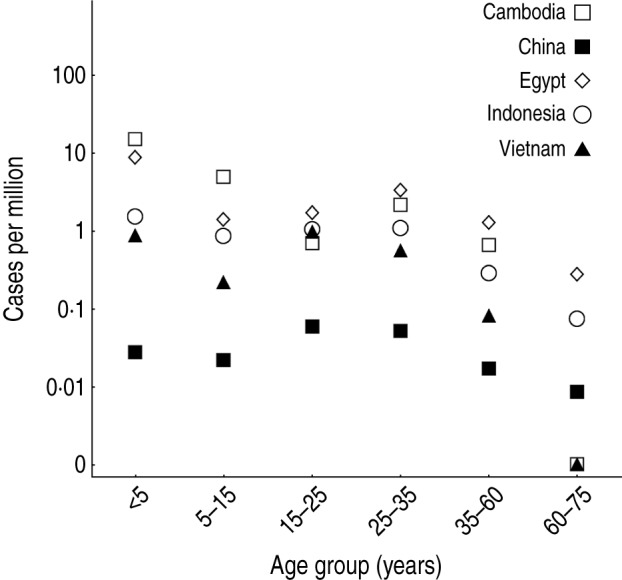
Demographic patterns of influenza A(H5N1) infection. Points show reported cases per million people, stratified by age group.

**Fig. 2.**
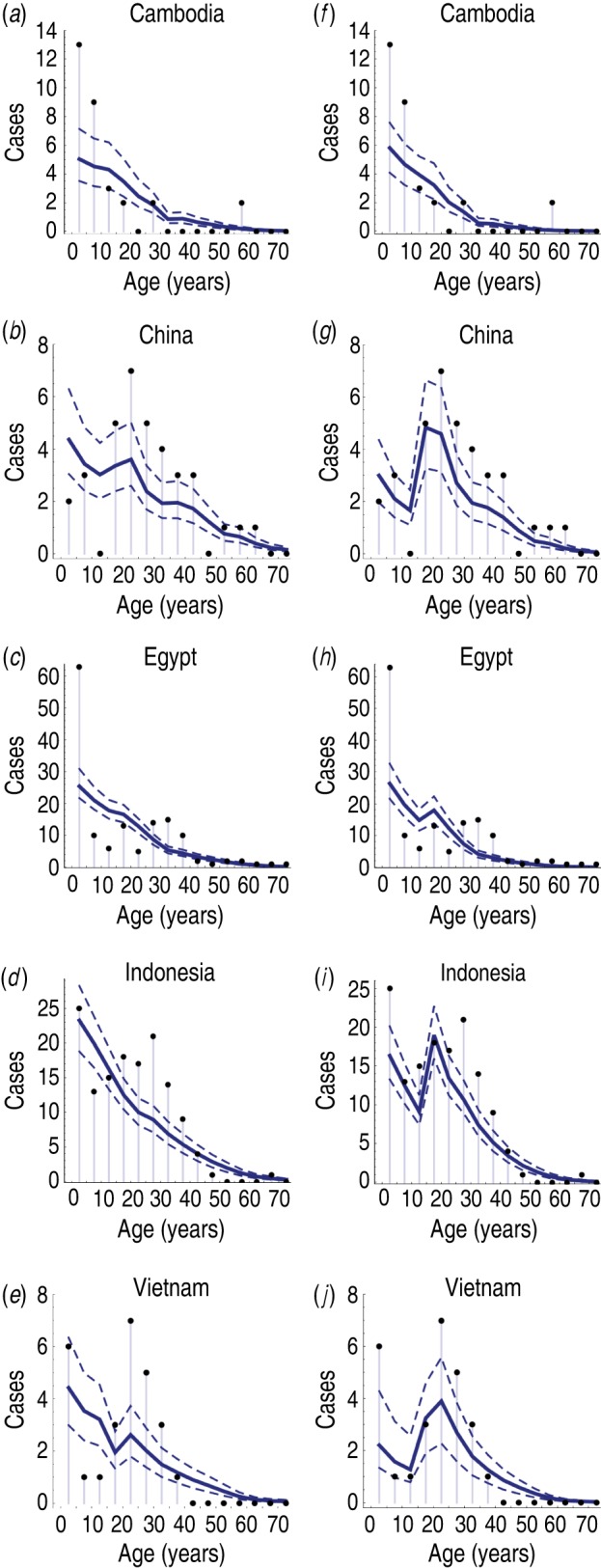
Comparison of reported cases in each country and model fits. (*a*–*e*) Results from model with cross-immunity only; (*f*–*j*) model with cross-immunity and age-dependent exposure risk. Dots show confirmed H5N1 cases in each 5-year age band; solid blue line shows model estimate; dashed lines give 95% credible intervals.

The age distribution of cases varied substantially between countries [3–5]. In most countries, there were a disproportionally high number of cases of influenza A(H5N1) in the <5 years age group, and another peak in the 25–35 years age group (Fig. 1). If reported spillover events occurred at an equal rate across all ages, we would expect the size of a particular age group to be predictive of the number of reported infections in that group. Hence the points in Figure 2*a* would form horizontal lines. However, it is possible that cross-immunity also contributed to the age distribution of reported cases. Influenza A(H1N1) and A(H5N1) viruses share the same neuraminidase subtype, N1, and there is evidence that H1N1 neuraminidase antibodies cross-react with H5N1 viruses [6–8].

To establish whether demographic profiles and heterosubtypic cross-immunity could explain observed patterns of infection, we used a mechanistic model to measure how spillover risk combines with cross-immunity from prior infection in the host population. We also analysed the relationship between exposure and infection, and examined the extent to which age-specific variation in risk of H5N1 transmission – with or without additional cross-immunity from H1N1 infection – could have shaped the observed distribution of H5N1 infection.

## METHODS

We compiled a line list of confirmed cases of influenza A(H5N1) reported to the World Health Organization between 1 January 2006 and 1 December 2013. For each case we gathered details of location and age of patient. Demographic data for each country was obtained from the United Nations Statistics Division Demographic Statistics database.

We assumed that the number of reported cases in each age group followed a Poisson distribution. In our cross-immunity model, the expected number of cases depended on two things: spillover risk and cross-immunity from prior exposure to influenza A(H1N1). We assumed that the expected number of spillover events into each age group was equal to the population size (in millions) multiplied by a certain parameter. As we were fitting the model to total case numbers rather than temporal data, this parameter represented the total *per capita* number of spillover events rather than frequency of spillover.

To include cross-immunity, we assumed that the probability of having not yet been infected with influenza A(H1N1) decreased exponentially with age: the longer an individual is alive, the more opportunities they have had to become infected [9]. Given prior infection with H1N1, there was a certain probability that the host will subsequently have cross-immunity against H5N1. The expected number of reported H5N1 cases in each group was therefore equal to the number of spillover events multiplied by the number of people that have no cross-immunity from prior H1N1 infection. Hence our model for a specific age group was:
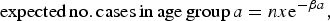
where *a* was the midpoint of the age group (i.e. 2·5 for the 0–5 years group), *β* was the average annual probability of gaining cross-immunity to H5N1 as a result of H1N1 infection, *n* was the number of individuals (in millions) and *x* was the number of spillover events per million people. There were six parameters in the model: *x* for each of the five countries, and *β*. We estimated these parameters using Markov chain Monte Carlo.

We also considered the possibility that age-specific infection patterns could be explained by age-dependent variation in risk of H5N1 exposure rather than cross-immunity. There were little data available on age-dependent patterns of contact with poultry and potential risk of exposure to H5N1 in the five countries we considered. However, one cross-sectional survey in Cambodia suggested that individuals aged ⩾15 years had a higher H5N1 transmission risk potential than those aged <15 years [10].

To account for possible differences in exposure risk with age, we incorporated a step function into our basic spillover model, with different relative risks of H5N1 exposure for individuals aged <15 and ⩾15 years. We allowed the relative risk to vary for each country, with the constraint that in Cambodia those aged ⩾15 years had a larger risk than those aged <15 years (we imposed no constraints on the relative risk in other countries). Our model for a specific age group was therefore:
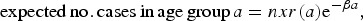
where *r*(*a*) denotes the relative risk of H5N1 exposure in individuals aged ⩾15 years, with *r*(*a*) = 1 if *a* < 15 and *r*(*a*) = *r* if *a*⩾15. If we set *β* = 0, we obtained a model with age-specific exposure risk only. In this model there were 10 parameters to estimate: *x* and *r* for each of the five countries. If we allow *β* to vary, there were 11 parameters to estimate.

Using the above models, we compared four different combinations of assumptions: no cross-immunity or age-specific variation in exposure; cross-immunity only; age-specific exposure only; or cross-immunity and age-specific exposure. We assessed model performance using the Bayesian Information Criterion (BIC) [11]. If *L* was the maximum likelihood estimate for a model with *p* parameters fitted to *d* data points:
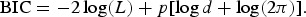
We compared candidate models by calculating ΔBIC, the difference between the model of interest and the smallest BIC of all models tested. If ΔBIC > 10 there is strong evidence against the model with the larger BIC. If ΔBIC < 2 there is a negligible difference between the two models [12].

## RESULTS

We found that models that included cross-immunity between influenza A(H1N1) and A(H5N1) performed much better than the others we considered (Table 1). The inclusion of age-specific influenza A(H5N1) exposure risk and cross-immunity produced a better fit as measured by log-likelihood, but the improvement in model performance did not provide clear evidence in favour of additional model complexity: there was negligible difference in ΔBIC between the two cross-immunity models. We also considered a model in which there were three different risk groups: <15, 15–60, and >60 years. However, even when cross-immunity is included, these models had less support than the original model with two risk groups (Table 2).

**Table 1.** Comparison of model performance

Model	Description	Parameters	Log likelihood	ΔBIC
1	Demography only	5	−235·9	106·9
2	Age-specific exposure	10	−219·3	104·3
3	Cross-immunity	6	−177·6	1·5
4	Age-specific exposure and cross-immunity	11	−161·5	0

BIC, Bayesian Information Criterion.

**Table 2.** Comparison of model performance when age-specific exposure is defined using a step function with two steps (age <15 years/⩾15 years) and three steps (age <15, 15–60, >60 years)

Model	Description	Parameters	Log likelihood	ΔBIC
1	Demography only	5	−235·9	106·9
2	Age-specific exposure (2 steps)	10	−219·3	104·3
3	Cross-immunity	6	−177·6	1·5
4	Age-specific exposure (2 steps) and cross-immunity	11	−161·5	0
5	Age-specific exposure (3 steps)	15	−211·1	126·4
6	Age-specific exposure (3 steps) and cross-immunity	16	−159·6	27·0

BIC, Bayesian Information Criterion.

Our parameter estimates suggest that spillover risk varied substantially between different countries. In the two best-performing models, the *per capita* number of spillover events was highest in Cambodia and Egypt, smaller in Indonesia and Vietnam, and lowest in China (Table 3). In all countries, the estimated number of spillover events was larger than the number of reported cases, suggesting that observed disease incidence would have been higher if host populations did not have pre-existing immunity. When the model included age-specific risk of exposure to H5N1 as well as cross-immunity, there was strong evidence that the risk was higher in the ⩾15 years age group in all countries (Table 4). In the best-performing model, which included both cross-immunity and age-specific exposure risk, we estimated that the annual probability of obtaining protection against H5N1 as a result of H1N1 infection was 0·06 [95% credible interval (CrI) 0·05–0·07]. In the cross-immunity only model, it was 0·04 (95% CrI 0·03–0·04).

**Table 3.** Estimated number of spillover events per million people for each country in the two best-performing models (3 and 4), and reported influenza A(H5N1) cases per million between 2006 and 2013

Country	Estimated spillover (model 3)	Estimated spillover (model 4)	Reported cases
Cambodia	4·83 (3·38–6·45)	7·19 (5·07–9·51)	2·35
China	0·08 (0·05–0·11)	0·16 (0·10–0·24)	0·03
Egypt	4·36 (3·55–5·41)	6·99 (5·55–9·34)	2·03
Indonesia	1·36 (1·06–1·69)	2·70 (2·13–3·53)	0·59
Vietnam	0·82 (0·54–1·20)	1·80 (1·11–2·81)	0·34

95% credible intervals are given in parentheses.

**Table 4.** Estimated relative influenza A(H5N1) exposure risk in individuals aged ⩾15 years compared to <15 years in each country between 2006 and 2013

Country	⩾15 years age group RR (95% CrI)
Cambodia	1·10 (1·00–1·50)
China	2·76 (2·07–3·43)
Egypt	1·44 (1·08–2·09)
Indonesia	3·03 (2·49–3·66)
Vietnam	5·23 (1·83–6·04)

RR, Relative risk; CrI, credible interval.

While the best-fitting models captured the general trend in reported cases with age, there were some discrepancies (Fig. 2). The number of reported H5N1 cases in the <5 years age group in Cambodia (Fig. 2*a, f*) and Egypt (Fig. 2*c, h*) was much higher than predicted by either model. It appears the surplus of H5N1 cases in the 20–40 years age groups in China, Indonesia and Vietnam could be partly explained by an increased risk of H5N1 exposure in these age groups (Fig. 2*g, i, j*).

## DISCUSSION

Using a simple mechanistic model, we examined the age distribution of reported influenza A(H5N1) cases in five countries between 2006 and 2013. Our analysis produced three key observations. First, much of the age pattern of infection can be explained through a combination of fixed spillover risk and age-dependent cross-immunity. In particular, the model reproduced the decline in reported cases with age in each country. Without cross-immunity, model performance was much worse, suggesting that demography alone cannot explain the observed age distribution of cases.

Second, there was a trade-off between age-specific cross-immunity and *per capita* spillover risk. Our estimates for spillover risk suggest that more than 50% of spillover events did not lead to reported infections, because hosts had partial immunity against H5N1 (Table 3). Such a discrepancy would not be immediately clear from the raw data presented in Figure 1.

Third, there was noticeable surplus of cases in individuals aged between 20 and 40 years, which were outside the 95% CrI of the simple cross-immunity model (Fig. 2*a–e*). Some of this variation could be explained when we used a model with age-specific H5N1 exposure risk (Fig. 2*f–j*), suggesting that H5N1 infection patterns depended on additional variables besides population demography and cross-immunity.

There are some limitations to our analysis. We used a simple model of infection, treating each country as a single population. In reality, there was likely to be regional variation in disease incidence [3, 4]. Further, we did not consider potential differences in reporting rate with age. Such differences could affect the number of reported spillover events, and hence explain some of the age-specific variation not captured by the model (Fig. 2).

As there were limited data available about the age-specific risk of H5N1 exposure, we used a simple step function to represent relative risk in different groups. This resulted in several additional model parameters, reducing model performance under BIC. Future empirical studies could help address this issue. By collecting data on factors that could affect H5N1 exposure risk, such as contact with animals, it would be possible to parameterize the age-dependent exposure risk directly. Such data would reduce model complexity, and could potentially provide a parsimonious explanation for the variation in influenza A(H5N1) infection patterns not captured by our model.

We estimated that the annual probability of gaining cross-immunity to H5N1 as a result of H1N1 infection was 0·04–0·06. A cohort study of 1793 participants in Vietnam reported 196 serologically confirmed influenza A(H1N1) infections over three seasons between 2007–2010 [13]. This suggests the average annual risk of H1N1 infection was 0·11 (95% CrI 0·09–0·12). If cross-immunity against H5N1 comes only from prior H1N1 infection, our estimates therefore suggest that infection with H1N1 has a 0·36–0·55 probability of conferring protection to H5N1. However, there is evidence that some individuals in Southeast Asia might have also had influenza A H5-specific antibodies from prior exposure to H5N1 [14]. These antibodies, or antibodies from prior exposure to other related viruses, could have contributed to cross-immunity as well. With population-level data on prior exposure to different subtypes, it might be possible to separate the relative contributions to immunity made by each subtype, and obtain better estimates for cross-immunity between influenza A(H1N1) and A(H5N1).

Our results suggest that cross-immunity and demography can explain several aspects of observed patterns of influenza A(H5N1) infection. Further investigation into cross-reaction between influenza subtypes could aid the interpretation of other infection data. For instance, cross-immunity might help explain why the age distribution of cases for H5N1 is much younger than for H7N9 [3, 15]: H5N1 shares a neuraminidase subtype with seasonal strains whereas H7N9 does not. In some instances, it might be necessary to consider both inter- and intra-subtyptic cross-immunity. There are concerns about the potential re-emergence of influenza A(H2N2) [16], a subtype that circulated in human populations between 1957 and 1968. However, older age groups might have immunity to H2N2 and/or cross-immunity from prior infection with A(H3N2).

Such cross-immunity is likely to influence influenza virus transmission [9]. When assessing potential control measures against emerging pathogens, is often assumed that the host population is fully susceptible to infection [17, 18]. This is the case for entirely novel pathogens, but for infections such as influenza A(H5N1), our results suggest it is important to account for potential cross-immunity when evaluating the virus's ability to spread in a human population.
